# Human- versus Artificial Intelligence

**DOI:** 10.3389/frai.2021.622364

**Published:** 2021-03-25

**Authors:** J. E. (Hans). Korteling, G. C. van de Boer-Visschedijk, R. A. M. Blankendaal, R. C. Boonekamp, A. R. Eikelboom

**Affiliations:** TNO Human Factors, Soesterberg, Netherlands

**Keywords:** human intelligence, artificial intelligence, artificial general intelligence, human-level artificial intelligence, cognitive complexity, narrow artificial intelligence, human-AI collaboration, cognitive bias

## Abstract

AI is one of the most debated subjects of today and there seems little common understanding concerning the differences and similarities of human intelligence and artificial intelligence. Discussions on many relevant topics, such as trustworthiness, explainability, and ethics are characterized by implicit anthropocentric and anthropomorphistic conceptions and, for instance, the pursuit of human-like intelligence as the golden standard for Artificial Intelligence. In order to provide more agreement and to substantiate possible future research objectives, this paper presents three notions on the similarities and differences between human- and artificial intelligence: 1) the fundamental constraints of human (and artificial) intelligence, 2) human intelligence as one of many possible forms of general intelligence, and 3) the high potential impact of multiple (integrated) forms of narrow-hybrid AI applications. For the time being, AI systems will have fundamentally different cognitive qualities and abilities than biological systems. For this reason, a most prominent issue is how we can use (and “collaborate” with) these systems as effectively as possible? For what tasks and under what conditions, decisions are safe to leave to AI and when is human judgment required? How can we capitalize on the specific strengths of human- and artificial intelligence? How to deploy AI systems effectively to complement and compensate for the inherent constraints of human cognition (and vice versa)? Should we pursue the development of AI “partners” with human (-level) intelligence or should we focus more at supplementing human limitations? In order to answer these questions, humans working with AI systems in the workplace or in policy making have to develop an adequate mental model of the underlying ‘psychological’ mechanisms of AI. So, in order to obtain well-functioning human-AI systems, *Intelligence Awareness* in humans should be addressed more vigorously. For this purpose a first framework for educational content is proposed.

## Introduction: Artificial and Human Intelligence, Worlds of Difference

### Artificial General Intelligence at the Human Level

Recent advances in information technology and in AI may allow for more coordination and integration between of humans and technology. Therefore, quite some attention has been devoted to the development of *Human-Aware* AI, which aims at AI that adapts as a “team member” to the cognitive possibilities and limitations of the human team members. Also metaphors like “mate,” “partner,” “alter ego,” “Intelligent Collaborator,” “buddy” and “mutual understanding” emphasize a high degree of collaboration, similarity, and equality in “hybrid teams”. When human-aware AI partners operate like “human collaborators” they must be able to sense, understand, and react to a wide range of complex human behavioral qualities, like attention, motivation, emotion, creativity, planning, or argumentation, (e.g. Krämer et al., 2012; van den Bosch and Bronkhorst, 2018; van den Bosch et al., 2019). Therefore these “AI partners,” or “team mates” have to be endowed with human-like (or humanoid) cognitive abilities enabling mutual understanding and collaboration (i.e. “human awareness”).

However, no matter how intelligent and autonomous AI agents become in certain respects, at least for the foreseeable future, they probably will remain unconscious machines or special-purpose devices that support humans in specific, complex tasks. As digital machines they are equipped with a completely different operating system (digital vs biological) and with correspondingly different cognitive qualities and abilities than biological creatures, like humans and other animals (Moravec, 1988; Klein et al., 2004; Korteling et al., 2018a; Shneiderman, 2020a). In general, digital reasoning- and problem-solving agents only compare very superficially to their biological counterparts, (e.g. Boden, 2017; Shneiderman, 2020b). Keeping that in mind, it becomes more and more important that human professionals working with advanced AI systems, (e.g. in military‐ or policy making teams) develop a proper mental model about the different cognitive capacities of AI systems in relation to human cognition. This issue will become increasingly relevant when AI systems become more advanced and are deployed with higher degrees of autonomy. Therefore, the present paper tries to provide some more clarity and insight into the fundamental characteristics, differences and idiosyncrasies of human/biological and artificial/digital intelligences. In the final section, a global framework for constructing educational content on this “Intelligence Awareness” is introduced. This can be used for the development of education and training programs for humans who have to use or “collaborate with” advanced AI systems in the near and far future.

With the application of AI systems with increasing autonomy more and more researchers consider the necessity of vigorously addressing the real complex issues of “human-level intelligence” and more broadly *artificial general intelligence*, or AGI, (e.g. Goertzel et al., 2014). Many different definitions of A(G)I have already been proposed, (e.g. Russell and Norvig, 2014 for an overview). Many of them boil down to: *technology containing or entailing (human-like) intelligence*, (e.g. Kurzweil, 1990). This is problematic. Most definitions use the term “intelligence”, as an essential element of the definition itself, which makes the definition tautological. Second, the idea that A(G)I should be *human-like* seems unwarranted. At least in natural environments there are many other forms and manifestations of highly complex and intelligent behaviors that are very different from specific *human* cognitive abilities (see Grind, 1997 for an overview). Finally, like what is also frequently seen in the field of biology, these A(G)I definitions use *human* intelligence as a central basis or analogy for reasoning about the—less familiar—phenomenon of A(G)I (Coley and Tanner, 2012). Because of the many differences between the underlying substrate and architecture of biological and artificial intelligence this *anthropocentric* way of reasoning is probably unwarranted. For these reasons we propose a (non-anthropocentric) definition of “intelligence” as: “*the capacity to realize complex goals*” (Tegmark, 2017). These goals may pertain to narrow, restricted tasks (narrow AI) or to broad task domains (AGI). Building on this definition, and on a definition of AGI proposed by Bieger et al. (2014) and one of Grind (1997), we define AGI here as: “*Non-biological capacities to autonomously and efficiently achieve complex goals in a wide range of environments”.* AGI systems should be able to identify and extract the most important features for their operation and learning process automatically and efficiently over a broad range of tasks and contexts. Relevant AGI research differs from the ordinary AI research by addressing the versatility and wholeness of intelligence, and by carrying out the engineering practice according to a system comparable to the human mind in a certain sense (Bieger et al., 2014).

It will be fascinating to create copies of ourselves which can learn iteratively by interaction with partners and thus become able to collaborate on the basis of common goals and mutual understanding and adaptation, (e.g.Bradshaw et al., 2012; Johnson et al., 2014). This would be very useful, for example when a high degree of social intelligence of AI will contribute to more adequate interactions with humans, for example in health care or for entertainment purposes (Wyrobek et al., 2008). True collaboration on the basis of common goals and mutual understanding necessarily implies some form of humanoid general intelligence. For the time being, this remains a goal on a far-off horizon. In the present paper we argue why for most applications it also may not be very practical or necessary (and probably a bit misleading) to vigorously aim or to anticipate on systems possessing “human-like” AGI or “human-like” abilities or qualities. The fact that humans possess general intelligence does not imply that new inorganic forms of general intelligence should comply to the criteria of human intelligence. In this connection, the present paper addresses the way we think about (natural and artificial) intelligence in relation to the most probable potentials (and real upcoming issues) of AI in the short- and mid-term future. This will provide food for thought in anticipation of a future that is difficult to predict for a field as dynamic as AI.

### What Is “Real Intelligence”?

Implicit in our aspiration of constructing AGI systems possessing humanoid intelligence is the premise that human (general) intelligence is the “real” form of intelligence. This is even already implicitly articulated in the term “Artificial Intelligence”, as if it were not entirely real, i.e., real like non-artificial (biological) intelligence. Indeed, as humans we know ourselves as the entities with the highest intelligence ever observed in the Universe. And as an extension of this, we like to see ourselves as rational beings who are able to solve a wide range of complex problems under all kinds of circumstances using our experience and intuition, supplemented by the rules of logic, decision analysis and statistics. It is therefore not surprising that we have some difficulty to accept the idea that we might be a bit less smart than we keep on telling ourselves, i.e., “the next insult for humanity” (van Belkom, 2019). This goes as far that the rapid progress in the field of artificial intelligence is accompanied by a recurring redefinition of what should be considered “real (general) intelligence.” The conceptualization of intelligence, that is, the ability to autonomously and efficiently achieve complex goals, is then continuously adjusted and further restricted to: “those things that only humans can do.” In line with this, AI is then defined as “the study of how to make computers do things at which, at the moment, people are better” (Rich and Knight, 1991; Rich et al., 2009). This includes thinking of creative solutions, flexibly using contextual- and background information, the use of intuition and feeling, the ability to really “think and understand,” or the inclusion of emotion in an (ethical) consideration. These are then cited as the specific elements of *real* intelligence, (e.g. Bergstein, 2017). For instance, Facebook’s director of AI and a spokesman in the field, Yann LeCun, mentioned at a *Conference at MIT on the Future of Work* that machines are still far from having “the essence of intelligence.” That includes the ability to understand the physical world well enough to make predictions about basic aspects of it—to observe one thing and then use background knowledge to figure out what other things must also be true. Another way of saying this is that machines don’t have *common sense* (Bergstein, 2017), like submarines that cannot swim (van Belkom, 2019). When exclusive human capacities become our pivotal navigation points on the horizon we may miss some significant problems that may need our attention first.

To make this point clear, we first will provide some insight into the basic nature of both human and artificial intelligence. This is necessary for the substantiation of an adequate awareness of intelligence (*Intelligence Awareness*), and adequate research and education anticipating the development and application of A(G)I. For the time being, this is based on three essential notions that can (and should) be further elaborated in the near future.

• With regard to cognitive tasks, we are probably less smart than we think. So why should we vigorously focus on *human*-like AGI?
• Many different forms of intelligence are possible and general intelligence is therefore not necessarily the same as *humanoid* general intelligence (or “AGI on human level”).
• AGI is often not necessary; many complex problems can also be tackled effectively using multiple narrow AI’s.^1^

## We Are Probably Not so Smart as We Think

How intelligent are we actually? The answer to that question is determined to a large extent by the perspective from which this issue is viewed, and thus by the measures and criteria for intelligence that is chosen. For example, we could compare the nature and capacities of human intelligence with other animal species. In that case we appear highly intelligent. Thanks to our enormous learning capacity, we have by far the most extensive arsenal of cognitive abilities^2^ to autonomously solve complex problems and achieve complex objectives. This way we can solve a huge variety of arithmetic, conceptual, spatial, economic, socio-organizational, political, etc. problems. The primates—which differ only slightly from us in genetic terms—are far behind us in that respect. We can therefore legitimately qualify humans, as compared to other animal species that we know, as highly intelligent.

### Limited Cognitive Capacity

However, we can also look beyond this “*relative* interspecies perspective” and try to qualify our intelligence in more *absolute* terms, i.e., using a scale ranging from zero to what is physically possible. For example, we could view the computational capacity of a human brain as a physical system (Bostrom, 2014; Tegmark, 2017). The prevailing notion in this respect among AI scientists is that intelligence is ultimately a matter of information and computation, and (thus) not of flesh and blood and carbon atoms. In principle, there is no physical law preventing that physical systems (consisting of quarks and atoms, like our brain) can be built with a much greater computing power and intelligence than the human brain. This would imply that there is no insurmountable physical reason why machines one day cannot become much more intelligent than ourselves in all possible respects (Tegmark, 2017). Our intelligence is therefore *relatively* high compared to other animals, but in absolute terms it may be very limited in its physical computing capacity, albeit only by the limited size of our brain and its maximal possible number of neurons and glia cells, (e.g. Kahle, 1979).

To further define and assess our own (biological) intelligence, we can also discuss the evolution and nature of our biological thinking abilities. As a biological neural network of flesh and blood, necessary for survival, our brain has undergone an evolutionary optimization process of more than a billion years. In this extended period, it developed into a highly effective and efficient system for regulating essential biological functions and performing perceptive-motor and pattern-recognition tasks, such as gathering food, fighting and flighting, and mating. Almost during our entire evolution, the neural networks of our brain have been further optimized for these basic biological and perceptual motor processes that also lie at the basis of our daily practical skills, like cooking, gardening, or household jobs. Possibly because of the resulting proficiency for these kinds of tasks we may forget that these processes are characterized by extremely high *computational* complexity, (e.g. Moravec, 1988). For example, when we tie our shoelaces, many millions of signals flow in and out through a large number of different sensor systems, from tendon bodies and muscle spindles in our extremities to our retina, otolithic organs and semi-circular channels in the head, (e.g. Brodal, 1981). This enormous amount of information from many different perceptual-motor systems is continuously, parallel, effortless and even without conscious attention, processed in the neural networks of our brain (Minsky, 1986; Moravec, 1988; Grind, 1997). In order to achieve this, the brain has a number of universal (inherent) working mechanisms, such as association and associative learning (Shatz, 1992; Bar, 2007), potentiation and facilitation (Katz and Miledi, 1968; Bao et al., 1997), saturation and lateral inhibition (Isaacson and Scanziani, 2011; Korteling et al., 2018a).

These kinds of basic biological and perceptual-motor capacities have been developed and set down over many millions of years. Much later in our evolution—actually only very recently—our cognitive abilities and rational functions have started to develop. These cognitive abilities, or capacities, are probably less than 100 thousand years old, which may be qualified as “embryonal” on the time scale of evolution, (e.g. Petraglia and Korisettar, 1998; McBrearty and Brooks, 2000; Henshilwood and Marean, 2003). In addition, this very thin layer of human achievement has necessarily been built on these “ancient” neural intelligence for essential survival functions. So, our “higher” cognitive capacities are developed *from* and *with* these (neuro) biological regulation mechanisms (Damasio, 1994; Korteling and Toet, 2020). As a result, it should not be a surprise that the capacities of our brain for performing these recent cognitive functions are still rather limited. These limitations are manifested in many different ways, for instance:

‐The amount of cognitive information that we can consciously process (our working memory, span or attention) is very limited (Simon, 1955). The capacity of our working memory is approximately 10–50 bits per second (Tegmark, 2017).
‐Most cognitive tasks, like reading text or calculation, require our full attention and we usually need a lot of time to execute them. Mobile calculators can perform millions times more complex calculations than we can (Tegmark, 2017).
‐Although we can process lots of information in parallel, we cannot simultaneously execute cognitive tasks that require deliberation and attention, i.e., “multi-tasking” (Korteling, 1994; Rogers and Monsell, 1995; Rubinstein, Meyer, and Evans, 2001).
‐Acquired cognitive knowledge and skills of people (memory) tend to decay over time, much more than perceptual-motor skills. Because of this limited “retention” of information we easily forget substantial portions of what we have learned (Wingfield and Byrnes, 1981).

### Ingrained Cognitive Biases

Our limited processing capacity for cognitive tasks is not the only factor determining our cognitive intelligence. Except for an overall limited processing capacity, human cognitive information processing shows systematic distortions. These are manifested in many cognitive biases (Tversky and Kahneman, 1973, Tversky and Kahneman, 1974). Cognitive biases are systematic, universally occurring tendencies, inclinations, or dispositions that skew or distort information processes in ways that make their outcome inaccurate, suboptimal, or simply wrong, (e.g. Lichtenstein and Slovic, 1971; Tversky and Kahneman, 1981). Many biases occur in virtually the same way in many different decision situations (Shafir and LeBoeuf, 2002; Kahneman, 2011; Toet et al., 2016). The literature provides descriptions and demonstrations of over 200 biases. These tendencies are largely implicit and unconscious and feel quite naturally and self/evident when we are aware of these cognitive inclinations (Pronin et al., 2002; Risen, 2015; Korteling et al., 2018b). That is why they are often termed “intuitive” (Kahneman and Klein, 2009) or “irrational” (Shafir and LeBoeuf, 2002). Biased reasoning can result in quite acceptable outcomes in natural or everyday situations, especially when the time cost of reasoning is taken into account (Simon, 1955; Gigerenzer and Gaissmaier, 2011). However, people often deviate from rationality and/or the tenets of logic, calculation, and probability in inadvisable ways (Tversky and Kahneman, 1974; Shafir and LeBoeuf, 2002) leading to suboptimal decisions in terms of invested time and effort (costs) given the available information and expected benefits.

Biases are largely caused by *inherent* (or structural) characteristics and mechanisms of the brain as a neural network (Korteling et al., 2018a; Korteling and Toet, 2020). Basically, these mechanisms—such as association, facilitation, adaptation, or lateral inhibition—result in a modification of the original or available data and its processing, (e.g. weighting its importance). For instance, lateral inhibition is a universal neural process resulting in the magnification of differences in neural activity (contrast enhancement), which is very useful for perceptual-motor functions, maintaining physical integrity and allostasis, (i.e. biological survival functions). For these functions our nervous system has been optimized for millions of years. However, “higher” cognitive functions, like conceptual thinking, probability reasoning or calculation, have been developed only very recently in evolution. These functions are probably less than 100 thousand years old, and may, therefore, be qualified as “embryonal” on the time scale of evolution, (e.g. McBrearty and Brooks, 2000; Henshilwood and Marean, 2003; Petraglia and Korisettar, 2003). In addition, evolution could not develop these new cognitive functions from scratch, but instead had to build this embryonal, and thin layer of human achievement from its “ancient” neural heritage for the essential biological survival functions (Moravec, 1988). Since cognitive functions typically require exact calculation and proper weighting of data, data transformations—like lateral inhibition—may easily lead to systematic distortions, (i.e. biases) in cognitive information processing. Examples of the large number of biases caused by the inherent properties of biological neural networks are: Anchoring bias (biasing decisions toward previously acquired information, Furnham and Boo, 2011; Tversky and Kahneman, 1973, Tversky and Kahneman, 1974), the Hindsight bias (the tendency to erroneously perceive events as inevitable or more likely once they have occurred, Hoffrage et al., 2000; Roese and Vohs, 2012) the Availability bias (judging the frequency, importance, or likelihood of an event by the ease with which relevant instances come to mind, Tversky and Kahnemann, 1973; Tversky and Kahneman, 1974), and the Confirmation bias (the tendency to select, interpret, and remember information in a way that confirms one’s preconceptions, views, and expectations, Nickerson, 1998). In addition to these inherent (structural) limitations of (biological) neural networks, biases may also originate from functional evolutionary principles promoting the survival of our ancestors who, as hunter-gatherers, lived in small, close-knit groups (Haselton et al., 2005; Tooby and Cosmides, 2005). Cognitive biases can be caused by a mismatch between evolutionarily rationalized “heuristics” (“evolutionary rationality”: Haselton et al., 2009) and the current context or environment (Tooby and Cosmides, 2005). In this view, the same heuristics that optimized the chances of survival of our ancestors in their (natural) environment can lead to maladaptive (biased) behavior when they are used in our current (artificial) settings. Biases that have been considered as examples of this kind of mismatch are the Action bias (preferring action even when there is no rational justification to do this, Baron and Ritov, 2004; Patt and Zeckhauser, 2000), Social proof (the tendency to mirror or copy the actions and opinions of others, Cialdini, 1984), the Tragedy of the commons (prioritizing personal interests over the common good of the community, Hardin, 1968), and the Ingroup bias (favoring one’s own group above that of others, Taylor and Doria, 1981).

This hard-wired (neurally inherent and/or evolutionary ingrained) character of biased thinking makes it unlikely that simple and straightforward methods like training interventions or awareness courses will be very effective to ameliorate biases. This difficulty of bias mitigation seems indeed supported by the literature (Korteling et al., 2021).

## General Intelligence Is Not the Same as Human-like Intelligence

### Fundamental Differences Between Biological and Artificial Intelligence

We often think and deliberate about intelligence with an anthropocentric conception of our own intelligence in mind as an obvious and unambiguous reference. We tend to use this conception as a basis for reasoning about other, less familiar phenomena of intelligence, such as other forms of biological and artificial intelligence (Coley and Tanner, 2012). This may lead to fascinating questions and ideas. An example is the discussion about how and when the point of “intelligence at human level” will be achieved. For instance, Ackermann. (2018) writes: “Before reaching superintelligence, general AI means that a machine will have the same cognitive capabilities as a human being”. So, researchers deliberate extensively about the point in time when we will reach general AI, (e.g., Goertzel, 2007; Müller and Bostrom, 2016). We suppose that these kinds of questions are not quite on target. There are (in principle) many different possible types of (general) intelligence conceivable of which human-like intelligence is just one of those. This means, for example that the development of AI is determined by the constraint of physics and technology, and not by those of biological evolution. So, just as the intelligence of a hypothetical extraterrestrial visitor of our planet earth is likely to have a different (in-)organic structure with different characteristics, strengths, and weaknesses, than the human residents this will also apply to artificial forms of (general) intelligence. Below we briefly summarize a few fundamental differences between human and artificial intelligence (Bostrom, 2014):

‐Basic structure: Biological (carbon) intelligence is based on neural “wetware” which is fundamentally different from artificial (silicon-based) intelligence. As opposed to biological wetware, in silicon, or digital, systems “hardware” and “software” are independent of each other (Kosslyn and Koenig, 1992). When a biological system has learned a new skill, this will be bounded to the system itself. In contrast, if an AI system has learned a certain skill then the constituting algorithms can be directly copied to all other similar digital systems.
‐Speed: Signals from AI systems propagate with almost the speed of light. In humans, the conduction velocity of nerves proceeds with a speed of at most 120 m/s, which is extremely slow in the time scale of computers (Siegel and Sapru, 2005).
‐Connectivity and communication: People cannot directly communicate with each other. They communicate via language and gestures with limited bandwidth. This is slower and more difficult than the communication of AI systems that can be connected directly to each other. Thanks to this direct connection, they can also collaborate on the basis of integrated algorithms.
‐Updatability and scalability: AI systems have almost no constraints with regard to keep them up to date or to upscale and/or re-configure them, so that they have the right algorithms and the data processing and storage capacities necessary for the tasks they have to carry out. This capacity for rapid, structural expansion and immediate improvement hardly applies to people.
‐In contrast, biology does a lot with a little: organic brains are millions of times more efficient in energy consumption than computers. The human brain consumes less energy than a lightbulb, whereas a supercomputer with comparable computational performance uses enough electricity to power quite a village (Fischetti, 2011).

These kinds of differences in basic structure, speed, connectivity, updatability, scalability, and energy consumption will necessarily also lead to different qualities and limitations between human and artificial intelligence. Our response speed to simple stimuli is, for example, many thousands of times slower than that of artificial systems. Computer systems can very easily be connected directly to each other and as such can be part of one integrated system. This means that AI systems do not have to be seen as individual entities that can easily work alongside each other or have mutual misunderstandings. And if two AI systems are engaged in a task then they run a minimal risk to make a mistake because of miscommunications (think of autonomous vehicles approaching a crossroad). After all, they are intrinsically connected parts of the same system and the same algorithm (Gerla et al., 2014).

### Complexity and Moravec’s Paradox

Because biological, carbon-based, brains and digital, silicon-based, computers are optimized for completely different kinds of tasks (e.g., Moravec, 1988; Korteling et al., 2018b), human and artificial intelligence show fundamental and probably far-stretching differences. Because of these differences it may be very misleading to use our own mind as a basis, model or analogy for reasoning about AI. This may lead to erroneous conceptions, for example about the presumed abilities of humans and AI to perform complex tasks. Resulting flaws concerning information processing capacities emerge often in the psychological literature in which “complexity” and “difficulty” of tasks are used interchangeably (see for examples: Wood et al., 1987; McDowd and Craik, 1988). Task complexity is then assessed in an anthropocentric way, that is: by the degree to which we humans can perform or master it. So, we use the *difficulty* to perform or master a task as a measure of its *complexity*, and task performance (speed, errors) as a measure of skill and intelligence of the task performer. Although this could sometimes be acceptable in psychological research, this may be misleading if we strive for understanding the intelligence of AI systems. For us it is much more difficult to multiply two random numbers of six digits than to recognize a friend on a photograph. But when it comes to counting or arithmetic operations, computers are thousands of times faster and better, while the same systems have only recently taken steps in image recognition (which only succeeded when deep learning technology, based on some principles of biological neural networks, was developed). In general: cognitive tasks that are relatively difficult for the human brain (and which we therefore find subjectively difficult) do not have to be computationally complex, (e.g., in terms of objective arithmetic, logic, and abstract operations). And vice versa: tasks that are relatively easy for the brain (recognizing patterns, perceptual-motor tasks, well-trained tasks) do not have to be computationally simple. This phenomenon, that which is easy for the ancient, neural “technology” of people and difficult for the modern, digital technology of computers (and vice versa) has been termed the *moravec’s Paradox.* Hans Moravec (1988) wrote: “It is comparatively easy to make computers exhibit adult level performance on intelligence tests or playing checkers, and difficult or impossible to give them the skills of a one-year-old when it comes to perception and mobility.”

### Human Superior Perceptual-Motor Intelligence

Moravec’s paradox implies that biological neural networks are intelligent in different ways than artificial neural networks. Intelligence is not limited to the problems or goals that we as humans, equipped with biological intelligence, find difficult (Grind, 1997). Intelligence, defined as the ability to realize complex goals or solve complex problems, is much more than that. According to Moravec (1988) high-level reasoning requires very little computation, but low-level perceptual-motor skills require enormous computational resources. If we express the complexity of a problem in terms of the number of elementary calculations needed to solve it, then our biological perceptual motor intelligence is *highly superior* to our cognitive intelligence. Our organic perceptual-motor intelligence is especially good at associative processing of higher-order invariants (“patterns”) in the ambient information. These are computationally more complex and contain more information than the simple, individual elements (Gibson, 1966, Gibson, 1979). An example of our superior perceptual-motor abilities is the *Object Superiority Effect*: we perceive and interpret whole objects faster and more effective than the (more simple) individual elements that make up these objects (Weisstein and Harris, 1974; McClelland, 1978; Williams and Weisstein, 1978; Pomerantz, 1981). Thus, letters are also perceived more accurately when presented as part of a word than when presented in isolation, i.e. the Word superiority effect, (e.g. Reicher, 1969; Wheeler, 1970). So, the *difficulty* of a task does not necessarily indicate its inherent *complexity*. As Moravec (1988) puts it: “We are all prodigious Olympians in perceptual and motor areas, so good that we make the difficult look easy. Abstract thought, though, is a new trick, perhaps less than 100 thousand years old. We have not yet mastered it. It is not all that intrinsically difficult; it just seems so when we do it.”

### The Supposition of Human-like AGI

So, if there would exist AI systems with general intelligence that can be used for a wide range of complex problems and objectives, those AGI machines would probably have a completely different intelligence profile, including other cognitive qualities, than humans have (Goertzel, 2007). This will be even so, if we manage to construct AI agents who display similar behavior like us and if they are enabled to adapt to our way of thinking and problem-solving in order to promote human-AI teaming. Unless we decide to deliberately *degrade* the capabilities of AI systems (which would not be very smart), the underlying capacities and abilities of man and machines with regard to collection and processing of information, data analysis, probability reasoning, logic, memory capacity etc. will still remain dissimilar. Because of these differences we should focus at systems that effectively *complement* us, and that make the human-AI system stronger and more effective. Instead of pursuing human-level AI it would be more beneficial to focus on autonomous machines and (support) systems that fill in, or extend on, the manifold gaps of human cognitive intelligence. For instance, whereas people are forced—by the slowness and other limitations of biological brains—to think heuristically in terms of goals, virtues, rules and norms expressed in (fuzzy) language, AI has already established excellent capacities to process and calculate directly on highly complex data. Therefore, or the execution of specific (narrow) cognitive tasks (logical, analytical, computational), modern digital intelligence may be more effective and efficient than biological intelligence. AI may thus help to produce better answers for complex problems using high amounts of data, consistent sets of ethical principles and goals, probabilistic-, and logic reasoning, (e.g. Korteling et al., 2018b). Therefore, we conjecture that ultimately the development of AI systems for supporting human decision making may appear the most effective way leading to the making of better choices or the development of better solutions on complex issues. So, the cooperation and division of tasks between people and AI systems will have to be primarily determinated by their mutually specific qualities. For example, tasks or task components that appeal to capacities in which AI systems excel, will have to be less (or less fully) mastered by people, so that less training will probably be required. AI systems are already much better than people at logically and arithmetically correct gathering (selecting) and processing (weighing, prioritizing, analyzing, combining) large amounts of data. They do this quickly, accurately and reliably. They are also more stable (consistent) than humans, have no stress and emotions and have a great perseverance and a much better retention of knowledge and skills than people. As a machine, they serve people completely and without any “self-interest” or “own hidden agenda.” Based on these qualities AI systems may effectively take over tasks, or task components, from people. However, it remains important that people continue to master those tasks to a certain extent, so that they can take over tasks or take adequate action if the machine system fails.

In general, people are better suited than AI systems for a much broader spectrum of cognitive and social tasks under a wide variety of (unforeseen) circumstances and events (Korteling et al., 2018b). People are also better at the social-psychosocial interaction for the time being. For example, it is difficult for AI systems to interpret human language and -symbolism. This requires a very extensive frame of reference, which, at least until now and for the near future, is difficult to achieve within AI. As a result of all these differences, people are still better at responding (as a flexible team) to unexpected and unpredictable situations and creatively devising possibilities and solutions in open and ill-defined tasks and across a wide range of different, and possibly unexpected, circumstances. People will have to make extra use of their specific human qualities, (i.e. what people are relatively good at) and train to improve relevant competencies. In addition, human team members will have to learn to deal well with the overall limitations of AIs. With such a proper division of tasks, capitalizing on the specific qualities and limitations of humans and AI systems, human decisional biases may be circumvented and better performance may be expected. This means that enhancement of a team with intelligent machines having less cognitive constraints and biases, may have more surplus value than striving at collaboration between humans and AI that have developed the same (human) biases. Although cooperation in teams with AI systems may need extra training in order to effectively deal with this bias-mismatch, this heterogeneity will probably be better and safer. This also opens up the possibility of a combination of high levels of meaningful human control AND high levels of automation which is likely to produce the most effective and safe human-AI systems (Elands et al., 2019; Shneiderman, 2020a). In brief: human intelligence is not the golden standard for general intelligence; instead of aiming at *human-like* AGI, the pursuit of AGI should thus focus on effective *digital/silicon AGI* in conjunction with an optimal configuration and allocation of tasks.

### Explainability and Trust

Developments in relation to artificial learning, or deep (reinforcement) learning, in particular have been revolutionary. Deep learning simulates a network resembling the layered neural networks of our brain. Based on large quantities of data, the network learns to recognize patterns and links to a high level of accuracy and then connect them to courses of action without knowing the underlying causal links. This implies that it is difficult to provide deep learning AI with some kind of transparency in how or why it has made a particular choice by, for example, by expressing an intelligible reasoning (for humans) about its decision process, like we do, (e.g. Belkom, 2019). In addition, reasoning about decisions like humans do is a very malleable and ad hoc process (at least in humans). Humans are generally unaware of their implicit cognitions or attitudes, and therefore not be able to adequately report on them. It is therefore rather difficult for many humans to introspectively analyze their mental states, as far as these are conscious, and attach the results of this analysis to verbal labels and descriptions, (e.g. Nosek et al. (2011). First, the human brain hardly reveals how it creates conscious thoughts, (e.g. Feldman-Barret, 2017). What it actually does is giving us the illusion that its products reveal its inner workings. In other words: our conscious thoughts tell us nothing about the way in which these thoughts came about. There is also no subjective marker that distinguishes correct reasoning processes from erroneous ones (Kahneman and Klein, 2009). The decision maker therefore has no way to distinguish between correct thoughts, emanating from genuine knowledge and expertize, and incorrect ones following from inappropriate neuro-evolutionary processes, tendencies, and primal intuitions. So here we could ask the question: isn’t it more trustworthy to have a real black box, than to listen to a confabulating one? In addition, according to Werkhoven et al. (2018) demanding explainability observability, or transparency (Belkom, 2019; van den Bosch et al., 2019) may cause artificial intelligent systems to constrain their potential benefit for human society, to what can be understood by humans.

Of course we should not blindly trust the results generated by AI. Like other fields of complex technology, (e.g. Modeling & Simulation), AI systems need to be verified (meeting specifications) and validated (meeting the systems’ goals) with regard to the objectives for which the system was designed. In general, when a system is properly verified and validated, it may be considered safe, secure and fit for purpose. It therefore deserves our trust for (logically) comprehensible and objective reasons (although mistakes still can happen). Likewise people trust in the performance of aero planes and cell phones despite we are almost completely ignorant about their complex inner processes. Like our own brains, artificial neural networks are fundamentally intransparant (Nosek et al., 2011; Feldman-Barret, 2017). Therefore, trust in AI should be primarily based on its objective performance. This forms a more important base than providing trust on the basis of subjective (trickable) impressions, stories, or images aimed at belief and appeal to the user. Based on empirical validation research, developers and users can explicitly verify how well the system is doing with respect to the set of values and goals for which the machine was designed. At some point, humans may want to trust that goals can be achieved against less cost and better outcomes, when we accept solutions even if they may be less transparent for humans (Werkhoven et al., 2018).

## The Impact of Multiple Narrow AI Technology

### AGI as the Holy Grail

AGI, like human general intelligence, would have many obvious advantages, compared to narrow (limited, weak, specialized) AI. An AGI system would be much more flexible and adaptive. On the basis of generic training and reasoning processes it would understand autonomously how multiple problems in all kinds of different domains can be solved in relation to their context, (e.g. Kurzweil, 2005). AGI systems also require far fewer human interventions to accommodate the various loose ends among partial elements, facets, and perspectives in complex situations. AGI would really understand problems and is capable to view them from different perspectives (as people—ideally—also can do). A characteristic of the current (narrow) AI tools is that they are skilled in a very specific task, where they can often perform at superhuman levels, (e.g. Goertzel, 2007; Silver et al., 2017). These specific tasks have been well-defined and structured. Narrow AI systems are less suitable, or totally unsuitable, for tasks or task environments that offer little structure, consistency, rules or guidance, in which all sorts of unexpected, rare or uncommon events, (e.g. emergencies) may occur. Knowing and following fixed procedures usually does not lead to proper solutions in these varying circumstances. In the context of (unforeseen) changes in goals or circumstances, the adequacy of current AI is considerably reduced because it cannot reason from a general perspective and adapt accordingly (Lake et al., 2017; Horowitz, 2018). As with narrow AI systems, people are then needed to supervise on these deviations in order to enable flexible and adaptive system performance. Therefore the quest of AGI may be considered as looking for a kind of holy grail.

### Multiple Narrow AI is Most Relevant Now!

The potential high prospects of AGI, however, do not imply that AGI will be the most crucial factor in future AI R&D, at least for the short- and mid-term. When reflecting on the great potential benefits of general intelligence, we tend to consider narrow AI applications as separate entities that can very well be outperformed by a broader AGI that presumably can deal with everything. But just as our modern world has evolved rapidly through a diversity of specific (limited) technological innovations, at the system level the total and wide range of emerging AI applications will also have a groundbreaking technological and societal impact (Peeters et al., 2020). This will be all the more relevant for the future world of big data, in which everything is connected to everything through the *Internet of Things*. So, it will be much more profitable and beneficial to develop and build (non-human-like) AI variants that will excel in areas where people are inherently limited. It seems not too far-fetched to suppose that the multiple variants of narrow AI applications also gradually get more broadly interconnected. In this way, a development toward an ever broader realm of integrated AI applications may be expected. In addition, it is already possible to train a language model AI (Generative Pre-trained Transformer3, GPT-3) with a gigantic dataset and then have it learn various tasks based on a handful of examples—one or few-shot learning. GPT-3 (developed by OpenAI) can do this with language-related tasks, but there is no reason why this should not be possible with image and sound, or with combinations of these three (Brown, 2020).

Besides, the moravec Paradox implies that the development of AI “partners” with many kinds of human (-level) qualities will be very difficult to obtain, whereas their added value, (i.e. beyond the boundaries of human capabilities) will be relatively low. The most fruitful AI applications will mainly involve supplementing human constraints and limitations. Given the present incentives for competitive technological progress, multiple forms of (connected) narrow AI systems will be the major driver of AI impact on our society for short- and mid-term. For the near future, this may imply that AI applications will remain very different from, and in many aspects almost incomparable with, human agents. This is likely to be true even if the hypothetical match of artificial general intelligence (AGI) with human cognition were to be achieved in the future in the longer term. Intelligence is a multi-dimensional (quantitative, qualitative) concept. All dimensions of AI unfold and grow along their own different path with their own dynamics. Therefore, over time an increasing number of specific (narrow) AI capacities may gradually match, overtake and transcend human cognitive capacities. Given the enormous advantages of AI, for example in the field of data availability and data processing capacities, the realization of AGI probably would at the same time outclass human intelligence in many ways. Which implies that the hypothetical point of time of matching human- and artificial cognitive capacities, i.e. human-level AGI, will probably be hard to define in a meaningful way (Goertzel, 2007).^3^

So when AI will truly understand us as a “friend,” “partner,” “alter ego” or “buddy,” as we do when we collaborate with other humans as humans, it will surpass us in many areas at the same Moravec (1998) time. It will have a completely different profile of capacities and abilities and thus it will not be easy to really understand the way it “thinks” and comes to its decisions. In the meantime, however, as the capacities of robots expand and move from simple tools to more integrated systems, it is important to calibrate our expectations and perceptions toward robots appropriately. So, we will have to enhance our awareness and insight concerning the continuous development and progression of multiple forms of (integrated) AI systems. This concerns for example the multi-facetted nature of intelligence. Different kind of agents may have different combinations of intelligences of very different levels. An agent with general intelligence may for example be endowed with excellent abilities on the area of image recognition and navigation, calculation, and logical reasoning while at the same time being dull on the area of social interaction and goal-oriented problem solving. This awareness of the multi-dimensional nature of intelligence also concerns the way we have to deal with (*and* capitalize on) anthropomorphism. That is the human tendency in human-robot interaction to characterize non-human artifacts that superficially look similar to us as possessing human-like traits, emotions, and intentions, (e.g., Kiesler and Hinds, 2004; Fink, 2012; Haring et al., 2018). Insight into these human factors issues is crucial to optimize the utility, performance and safety of human-AI systems (Peeters et al., 2020).

From this perspective, the question whether or not “AGI at the human level” will be realized is not the most relevant question for the time being. According to most AI scientists, this will certainly happen, and the key question is not IF this will happen, but WHEN, (e.g., Müller and Bostrom, 2016). At a system level, however, multiple narrow AI applications are likely to overtake human intelligence in an increasingly wide range of areas.

## Conclusions and Framework

The present paper focused on providing some more clarity and insight into the fundamental characteristics, differences and idiosyncrasies of human and artificial intelligences. First we presented ideas and arguments to scale up and differentiate our conception of intelligence, whether this may be human or artificial. Central to this broader, multi-faceted, conception of intelligence is the notion that intelligence in itself is a matter of information and computation, independent of its physical substrate. However, the nature of this physical substrate (biological/carbon or digital/silicon), will substantially determine its potential envelope of cognitive abilities and limitations. Organic cognitive faculties of humans have been very recently developed during the evolution of mankind. These “embryonal” faculties have been built on top of a biological neural network apparatus that has been optimized for allostasis and (complex) perceptual motor functions. Human cognition is therefore characterized by various structural limitations and distortions in its capacity to process certain forms of non-biological information. Biological neural networks are, for example, not very capable of performing arithmetic calculations, for which my pocket calculator fits millions of times better. These inherent and ingrained limitations, that are due to the biological and evolutionary origin of human intelligence, may be termed “hard-wired.”

In line with the *Moravic’s paradox*, we argued that intelligent behavior is more than what *we, as homo sapiens,* find difficult. So we should not confuse task-difficulty (subjective, anthropocentric) with task-complexity (objective). Instead we advocated a versatile conceptualization of intelligence and an acknowledgment of its many possible forms and compositions. This implies a high variety in types of biological or other forms of high (general) intelligence with a broad range of possible intelligence profiles and cognitive qualities (which may or may not surpass ours in many ways). This would make us better aware of the most probable potentials of AI applications for the short- and medium-term future. For example, from this perspective, our primary research focus should be on those components of the intelligence spectrum that are relatively difficult for the human brain and relatively easy for machines. This involves primarily the *cognitive* component requiring calculation, arithmetic analysis, statistics, probability calculation, data analysis, logical reasoning, memorization, et cetera.

In line with this we have advocated a modest, more humble, view of our human, general intelligence. Which also implies that human-level AGI should not be considered as the “golden standard” of intelligence (to be pursued with foremost priority). Because of the many fundamental differences between natural and artificial intelligences, human-like AGI will be very difficult to accomplish in the first place (and also with relatively limited added value). In case an AGI will be accomplished in the (far) future it will therefore probably have a completely different profile of cognitive capacities and abilities than we, as humans, have. When such an AGI has come so far that it is able to “collaborate” like a human, it will at the same time be likely that can in many respects already function at highly superior levels relative to what we are able to. For the time being, however, it will not be very realistic and useful to aim at AGI that includes the broad scope of human perceptual-motor and cognitive abilities. Instead, the most profitable AI applications for the short- and mid-term future, will probably be based on multiple narrow AI systems. These multiple narrow AI applications may catch up with human intelligence in an increasingly broader range of areas.

From this point of view we advocate not to dwell too intensively on the AGI question, whether or when AI will outsmart us, take our jobs, or how to endow it with all kinds of human abilities. Given the present state of the art it may be wise to focus more on the whole system of multiple AI innovations with humans as a crucial connecting and supervising factor. This also implies the establishment and formalization of legal boundaries and proper (effective, ethical, safe) goals for AI systems (Elands et al., 2019; Aliman, 2020). So this human factor (legislator, user, “collaborator”) needs to have good insight into the characteristics and capacities of biological and artificial intelligence (under all sorts of tasks and working conditions). Both in the workplace and in policy making the most fruitful AI applications will be to complement and compensate for the inherent biological and cognitive constraints of humans. For this reason, prominent issues concern how to use it intelligently? For what tasks and under what conditions decisions are safe to leave to AI and when is human judgment required? How can we capitalize on the strengths of human intelligence and how to deploy AI systems effectively to complement and compensate for the inherent constraints of human cognition. See (Hoffman and Johnson, 2019; Shneiderman, 2020a; Shneiderman, 2020b) for recent overviews.

In summary: No matter how intelligent autonomous AI agents become in certain respects, at least for the foreseeable future, they will remain unconscious machines. These machines have a fundamentally different operating system (biological vs digital) with correspondingly different cognitive abilities and qualities than people and other animals. So, before a proper “team collaboration” can start, the human team members will have to understand these kinds of differences, i.e., how human information processing and intelligence differs from that of–the many possible and specific variants of—AI systems. Only when humans develop a proper of these “interspecies” differences they can effectively capitalize on the potential benefits of AI in (future) human-AI teams. Given the high flexibility, versatility, and adaptability of humans relative to AI systems, the first challenge becomes then how to ensure human adaptation to the more rigid abilities of AI?^4^ In other words: how can we achieve a proper conception the differences between human- and artificial intelligence?

### Framework for Intelligence Awareness Training

For this question, the issue of *Intelligence Awareness* in human professionals needs to be addressed more vigorously. Next to computer tools for the distribution of relevant awareness information (Collazos et al., 2019) in human-machine systems, this requires better education and training on how to deal with the very new and different characteristics, idiosyncrasies, and capacities of AI systems. This includes, for example, a proper understanding of the basic characteristics, possibilities, and limitations of the AI’s cognitive system properties without anthropocentric and/or anthropomorphic misconceptions. In general, this *“Intelligence Awareness”* is highly relevant in order to better understand, investigate, and deal with the manifold possibilities and challenges of machine intelligence. This practical human-factors challenge could, for instance, be tackled by developing new, targeted and easily configurable (adaptive) training forms and learning environments for human-AI systems. These flexible training forms and environments, (e.g. simulations and games) should focus at developing knowledge, insight and practical skills concerning the specific, non-human characteristics, abilities, and limitations of AI systems and how to deal with these in practical situations. People will have to understand the critical factors determining the goals, performance, and choices of AI? Which may in some cases even include the simple notion that AIs excite as much about their performance in achieving their goals as your refrigerator does for keeping your milkshake well. They have to learn when and under what conditions decisions are safe to leave to AI and when is human judgment required or essential? And more in general: how does it “think” and decide? The relevance of this kind of knowledge, skills and practices will only become bigger when the degree of autonomy (and genericity) of advanced AI systems will grow.

What does such an *Intelligence Awareness* training curriculum look like? It needs to include at least a module on the cognitive characteristics of AI. This is basically a subject similar to those subjects that are also included in curricula on *human* cognition. This broad module on the “Cognitive Science of AI” may involve a range of sub-topics starting with a revision of the concept of "Intelligence" stripped of anthropocentric and anthropomorphic misunderstandings. In addition, this module should focus at providing knowledge about the structure and operation of the AI operating system or the “AI mind.” This may be followed by subjects like: Perception and interpretation of information by AI, AI cognition (memory, information processing, problem solving, biases), dealing with AI possibilities and limitations in the “human” areas like creativity, adaptivity, autonomy, reflection, and (self-) awareness, dealing with goal functions (valuation of actions in relation to cost-benefit), AI ethics and AI security. In addition, such a curriculum should include technical modules providing insight into the working of the AI operating system. Due to the enormous speed with which the AI technology and application develops, the content of such a curriculum is also very dynamic and continuously evolving on the basis of technological progress. This implies that the curriculum and training-aids and -environments should be flexible, experiential, and adaptive, which makes the work form of *serious gaming* ideally suited. Below, we provide a global framework for the development of new educational curricula on AI awareness. These subtopics go beyond learning to effectively “operate,” “control” or interact with specific AI applications (i.e. conventional human-machine interaction):

‐Understanding of underlying system characteristics of the AI (the “AI brain”). Understanding the specific qualities and limitations of AI relative to human intelligence.
‐Understanding the complexity of the tasks and of the environment from the perspective of AI systems.
‐Understanding the problem of biases in human cognition, relative to biases in AI.
‐Understanding the problems associated with the control of AI, predictability of AI behavior (decisions), building trust, maintaining situation awareness (complacency), dynamic task allocation, (e.g. taking over each other’s tasks) and responsibility (accountability).
‐How to deal with possibilities and limitations of AI in the field of “creativity”, adaptability of AI, “environmental awareness”, and generalization of knowledge.
‐Learning to deal with perceptual and cognitive limitations and possible errors of AI which may be difficult to comprehend.
‐Trust in the performance of AI (possibly in spite of limited transparency or ability to “explain”) based on verification and validation.
‐Learning to deal with our natural inclination to anthropocentrism and anthropomorphism (“theory of mind”) when reasoning about human-robot interaction.
‐How to capitalize on the powers of AI in order to deal with the inherent constraints of human information processing (and vice versa).
‐Understanding the specific characteristics and qualities of the man-machine system and being able to decide on when, for what, and how the integrated combination of human- and AI faculties may perform at best overall system potential.

In conclusion: due to the enormous speed with which the AI technology and application evolves we need a more versatile conceptualization of intelligence and an acknowledgment of its many possible forms and combinations. A revised conception of intelligence includes also a good understanding of the basic characteristics, possibilities, and limitations of different (biological, artificial) cognitive system properties without anthropocentric and/or anthropomorphic misconceptions. This “Intelligence Awareness” is highly relevant in order to better understand and deal with the manifold possibilities and challenges of machine intelligence, for instance to decide when to use or deploy AI in relation to tasks and their context. The development of educational curricula with new, targeted, and easily configurable training forms and learning environments for human-AI systems are therefore recommended. Further work should focus on training tools, methods and content that are flexible and adaptive enough to be able to keep up with the rapid changes in the field of AI and with the wide variety of target groups and learning goals.

## Footnotes

1 Narrow AI can be defined as the production of systems displaying intelligence regarding specific, highly constrained tasks, like playing chess, facial recognition, autonomous navigation, or locomotion (Goertzel et al., 2014).

2 Cognitive abilities involve deliberate, conceptual or analytic thinking (e.g., calculation, statistics, analysis, reasoning, abstraction)

3 Unless of course AI will be deliberately constrained or degraded to human-level functioning.

4 Next to the issue of Human-Aware AI, i.e. tuning AI to the cognitive characteristics of humans.
